# Long non-coding RNA levels can be modulated by 5-azacytidine in *Schistosoma mansoni*

**DOI:** 10.1038/s41598-020-78669-5

**Published:** 2020-12-09

**Authors:** Murilo S. Amaral, Lucas F. Maciel, Gilbert O. Silveira, Giovanna G. O. Olberg, João V. P. Leite, Lucas K. Imamura, Adriana S. A. Pereira, Patricia A. Miyasato, Eliana Nakano, Sergio Verjovski-Almeida

**Affiliations:** 1 Laboratório de Parasitologia, Instituto Butantan, São Paulo, Brazil; 2 Departamento de Bioquímica, Instituto de Química, Universidade de São Paulo, São Paulo, Brazil

**Keywords:** Long non-coding RNAs, Parasite biology

## Abstract

*Schistosoma mansoni* is a flatworm that causes schistosomiasis, a neglected tropical disease that affects more than 200 million people worldwide. There is only one drug indicated for treatment, praziquantel, which may lead to parasite resistance emergence. The ribonucleoside analogue 5-azacytidine (5-AzaC) is an epigenetic drug that inhibits *S. mansoni* oviposition and ovarian development through interference with parasite transcription, translation and stem cell activities. Therefore, studying the downstream pathways affected by 5-AzaC in *S. mansoni* may contribute to the discovery of new drug targets. Long non-coding RNAs (lncRNAs) are transcripts longer than 200 nucleotides with low or no protein coding potential that have been involved in reproduction, stem cell maintenance and drug resistance. We have recently published a catalog of lncRNAs expressed in *S. mansoni* life-cycle stages, tissues and single cells. However, it remains largely unknown if lncRNAs are responsive to epigenetic drugs in parasites. Here, we show by RNA-Seq re-analyses that hundreds of lncRNAs are differentially expressed after in vitro 5-AzaC treatment of *S. mansoni* females, including intergenic, antisense and sense lncRNAs. Many of these lncRNAs belong to co-expression network modules related to male metabolism and are also differentially expressed in unpaired compared with paired females and ovaries. Half of these lncRNAs possess histone marks at their genomic *loci*, indicating regulation by histone modification. Among a selected set of 8 lncRNAs, half of them were validated by RT-qPCR as differentially expressed in females, and some of them also in males. Interestingly, these lncRNAs are also expressed in other life-cycle stages. This study demonstrates that many lncRNAs potentially involved with *S. mansoni* reproductive biology are modulated by 5-AzaC and sheds light on the relevance of exploring lncRNAs in response to drug treatments in parasites.

## Introduction

Schistosomiasis is a very debilitating disease, spread across three continents with a global burden estimated by the World Health Organization at 2,543,364 DALYs (Disease Adjusted Life Years)^1^. It is estimated that schistosomiasis affects more than 200 million people in 74 countries^2,3^. The disease is caused by parasitic trematodes of the genus *Schistosoma*, being the three main species *Schistosoma mansoni*, *S. japonicum* and *S. haematobium*^4^. *S. mansoni* is the prevalent species in Latin America, with 1 to 3 million people infected and over 25 million living in risk areas mainly in Brazil and Venezuela^5^.

Administration of praziquantel (PZQ) to infected individuals is the basis of current schistosomiasis therapy. PZQ is a safe, cheap and tolerable drug^6^, however, cure rates of less than 50% have been recorded^7^ and drug tolerance has already been reported^8,9^. This scenario reinforces the need of new and more efficient approaches in reducing morbidity or disease eradication, such as the development of a vaccine^10^ or alternative drugs^11^.

5-azacytidine (5-AzaC) is a ribonucleoside currently used to treat human myelodysplastic syndrome (MDS) and acute myeloid leukemia (AML)^12^. 5-AzaC is considered an epigenetic drug as it can prevent DNA methylation by inhibition of DNA methyltransferases. It can also impede RNA methylation^13^ and decrease protein synthesis^14^. In *S. mansoni*, 5-AzaC has been shown to inhibit biological processes related to female metabolism, including egg production, egg maturation and normal ovarian development^15^. In addition, 5-AzaC also significantly alters *S. mansoni* adult female transcription, translation and stem cell activities^16^. Therefore, the study of the downstream pathways affected by 5-AzaC in *S. mansoni* may contribute to the understanding of the epigenetic control of gene expression and its physiological consequences in schistosomes and, in the future, to the possible development of new chemotherapeutic strategies against schistosomiasis.

Long non-coding RNAs (lncRNAs) are transcripts longer than 200 nucleotides with low or no protein coding potential^17,18^ that in humans are involved in a wide range of biological processes, including cell cycle regulation, reproduction, stem cell maintenance and drug resistance^19^. While the functions of lncRNAs have been explored^20^ and growing evidence suggests that they should be considered as drug targets in human diseases^21^, the mechanisms of regulation of lncRNA expression are much less understood^17^. In helminths other than *S. mansoni*, just a few works have reported the identification of lncRNAs using transcriptomic approaches^22–24^, however no further investigation of the mechanisms of lncRNA regulation or response to drug treatments were performed.

In *S. mansoni*, the expression of lncRNAs at different life-cycle stages was first detected by our group in 2011 using microarrays^25^ and then subsequently reported by many groups using RNA-Seq approaches^26–31^. However, it is largely unknown if *S. mansoni* lncRNA levels may be regulated by drugs. In the present work, we have evaluated the effect of 5-AzaC on lncRNA expression in *S. mansoni* adult worms by performing a re-analysis of the public RNA-Seq data from Geyer et al.^16^. We show, for the first time, that an epigenetic drug affects lncRNA levels in *S. mansoni* and that many of these lncRNAs are also differentially expressed in unpaired females and ovaries, indicating involvement in parasite reproductive biology. Understanding the mechanisms of control of lncRNAs expression will help the identification of potential new therapeutic targets and may contribute to the development of novel therapeutic strategies in the future.

## Results

### A set of lncRNAs is differentially expressed in *S. mansoni* females upon 5-AzaC in vitro treatment

We reanalyzed the RNA-Seq public data generated by Geyer et al.^16^ to search for long non-coding RNAs (lncRNAs) possibly affected by 5-AzaC treatment in *S. mansoni* females (Supplementary Table S1 shows the samples used and alignment statistics). In that study, Geyer et al.^16^ had treated *S. mansoni* adult worm pairs in vitro (Puerto Rican strain/NMRI, obtained from mice) with 5-AzaC at 491 μM for 48 h, extracted RNA from the females and then performed RNA-Seq. Thirty adult worm couples were used in each of three biological replicates^16^. Geyer et al.^16^ analyzed in that RNA-Seq dataset only the protein-coding genes differentially expressed in *S. mansoni* females after treatment with 5-AzaC. As lncRNA levels have been shown to be modulated by nucleoside analogs in other eukaryotes^32,33^, we hypothesized that lncRNA levels would also be modulated by 5-AzaC in *S. mansoni*.

**Figure 1:**
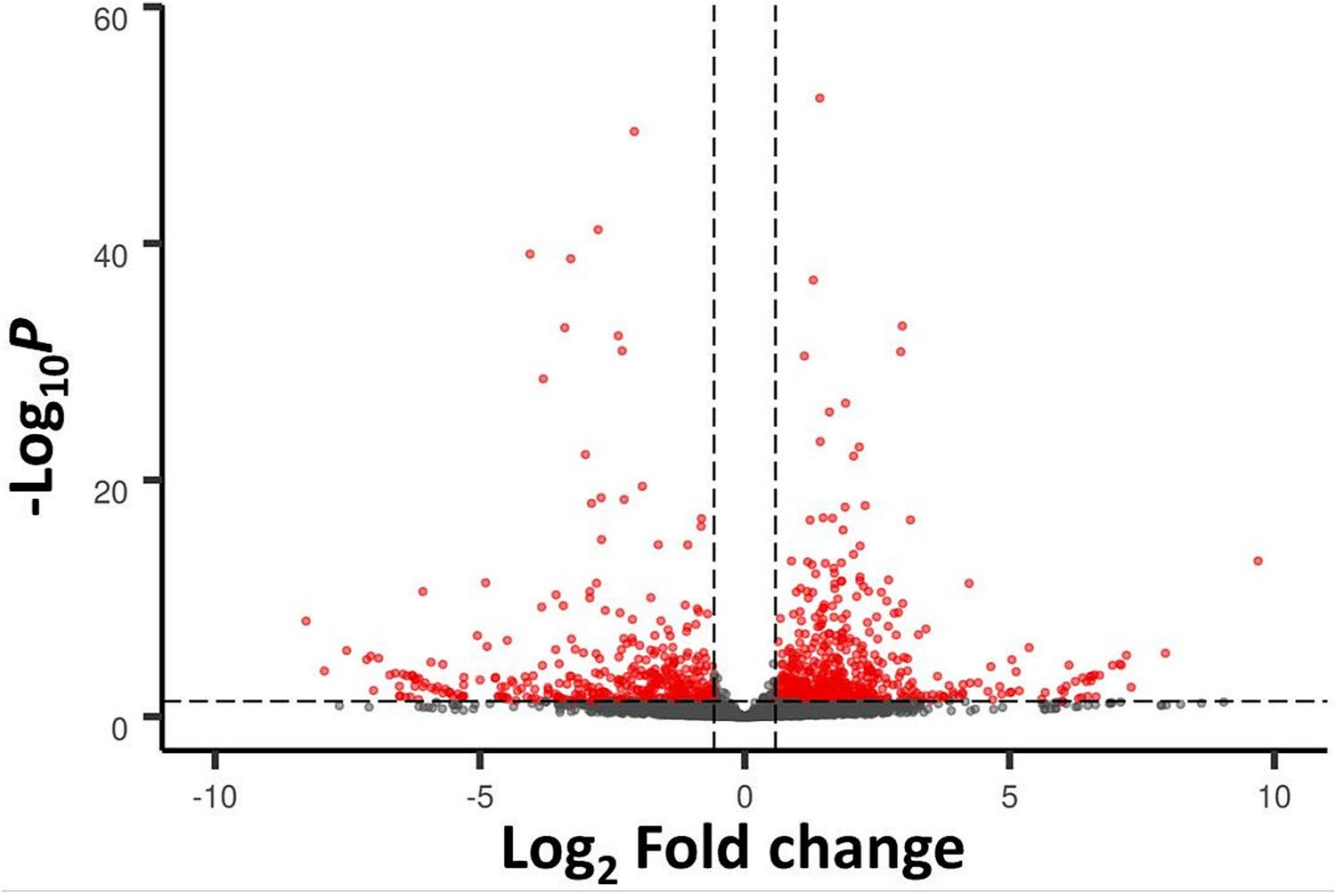
Differentially expressed long non-coding RNAs (lncRNAs) detected by RNA-Seq in adult *S. mansoni* females treated with 5-AzaC. These results were obtained by re-analysis of the RNA-Seq data from Geyer et al.^16^ using the *S. mansoni* lncRNA transcriptome published in Maciel et al.^31^ as reference. In Geyer et al.^16^, parasites were cultured either in the presence or absence of 491 μM 5-AzaC for 48 h. LncRNA gene expression levels identified in this RNA-Seq dataset are shown with a volcano plot, which displays the differentially expressed lncRNAs between 5-AzaC-treated and control *S. mansoni* females (red dots, showing FDR < 0.05 and log2FC > 0.59 or < -0.59, dotted lines). Grey dots represent non-differentially expressed lncRNAs. 912 lncRNAs were considered significantly differentially expressed, being 522 long intergenic ncRNAs, 358 antisense lncRNAs and 32 sense lncRNAs.

Indeed, the re-analysis of Geyer et al.^16^ RNA-Seq data with a reference transcriptome that is comprised of protein-coding genes as well as lncRNAs^31^ (see “Materials and methods”), found 912 lncRNAs differentially expressed in *S. mansoni* females upon 5-AzaC in vitro treatment. Among them, 522 were long intergenic non-coding RNAs (lincRNAs, being 353 upregulated and 169 downregulated), 358 were antisense non-coding RNAs (SmLNCAs, being 183 upregulated and 175 downregulated), and 32 were sense non-coding RNAs (SmLNCSs, being 16 upregulated and 16 downregulated) (Fig. 1). All differentially regulated protein-coding genes and lncRNAs are shown in Supplementary Table S2 with their transcript per million (TPM) values, and in Supplementary Table S3 with their raw counts.

We also found in our analysis 3219 protein-coding genes (corresponding to 3693 transcripts, 1655 upregulated and 2038 downregulated) differentially expressed after 5-AzaC treatment, of which 1810 have also been found as differentially expressed by Geyer et al.^16^. Geyer et al.^16^ previously identified 4036 protein-coding genes differentially expressed after 5-AzaC treatment, and 3221 of these genes are contained in the *S. mansoni* genome v7 annotation; thus, we were able to retrieve 1810 out of 3221 (or 56%) protein-coding genes found as differentially expressed in that work, which is a reasonable proportion considering the difference in the genomes used for reads alignment (genome version 7 was used here versus genome version 5.2 in the previous work) and the different read-mapping and counting tools used for the analysis.

As expected, principle component analysis (PCA) resulted in transcriptomes of the 5-AzaC treated and control groups segregating broadly into two distinct regions with replicates from the same condition clustering together, both for control or 5-AzaC-treated groups (Supplementary Fig. S1).

### Most of the lncRNAs differentially expressed upon 5-AzaC treatment in *S. mansoni* females belong to co-expression modules related to male metabolism

**Figure 2:**
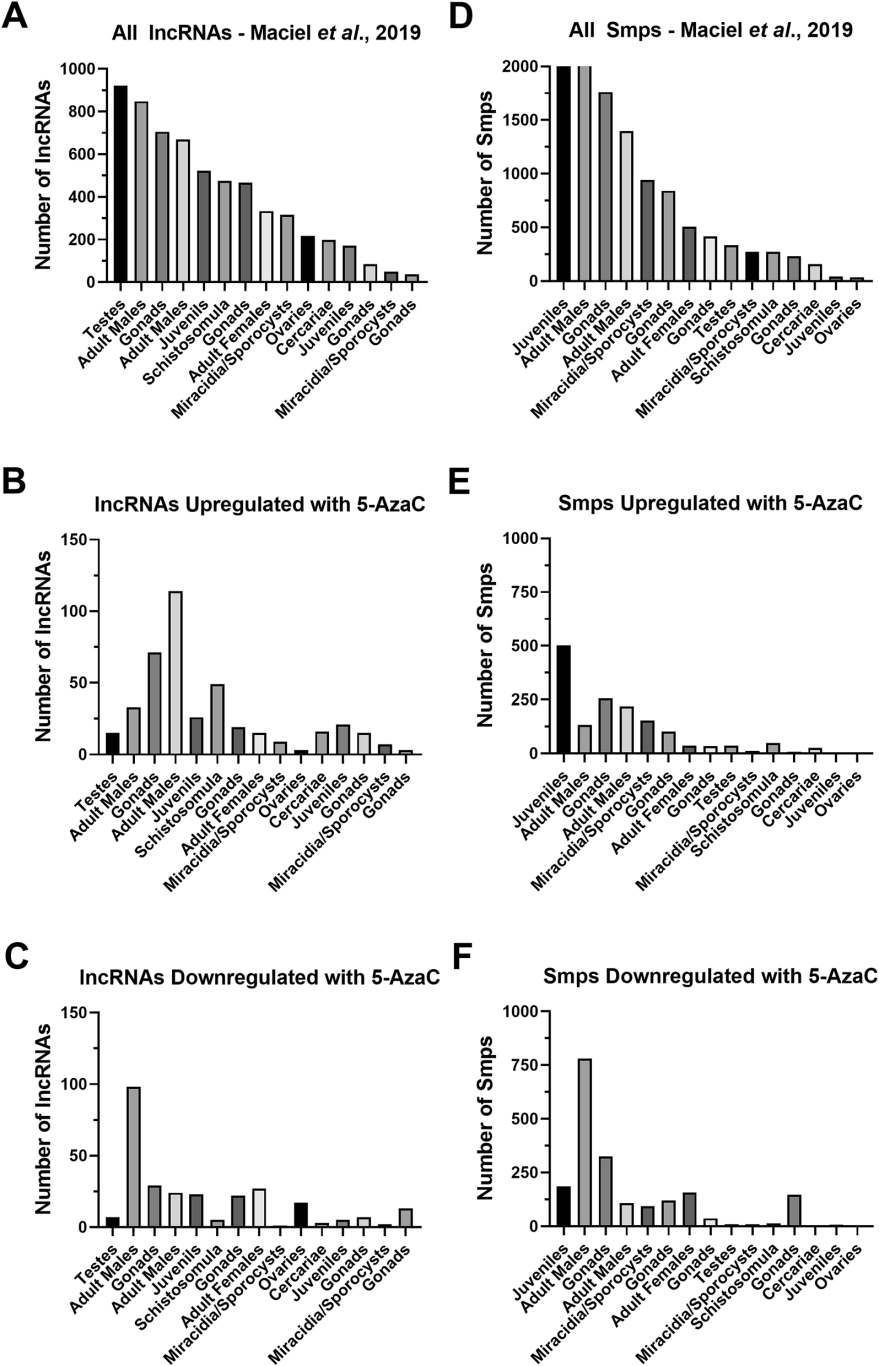
Distribution of 5-AzaC-affected long non-coding RNAs and protein-coding genes among the weighted gene co-expression network (WGCNA) modules (represented by life-cycle stages and tissues). (**A**) Number of lncRNAs detected in each of 15 different WGCNA modules, according to Maciel et al.^31^; note that different modules are associated to the same *S. mansoni* life-cycle stage/tissue. (**B**) Number of lncRNAs upregulated in 5-AzaC treated females in each of the 15 WGCNA modules. (**C**) Number of lncRNAs downregulated in 5-AzaC treated females in each of the 15 WGCNA modules. (**D**) Number of protein-coding genes detected in each of 15 different WGCNA modules, according to Maciel et al.^31^; note that different modules are associated to the same *S. mansoni* life-cycle stage/tissue. (**E**) Number of protein-coding genes upregulated in 5-AzaC treated females in each of the 15 WGCNA modules. (**F**) Number of protein-coding genes downregulated in 5-AzaC treated females in each of the 15 WGCNA modules.

Previously, besides building a new *S. mansoni* transcriptome comprised of lncRNAs in addition to protein-coding genes^31^, we also showed by weighted gene co-expression network analyses (WGCNA) that 6016 out of 16,583 lncRNAs identified in different *S. mansoni* life-cycle stages and tissues belong to one of 15 different lncRNAs/mRNAs co-expression modules^31^ (Fig. 2A). Each of these 15 modules represents one cluster of highly interconnected lncRNA/mRNA genes that are more expressed in one given *S. mansoni* stage/tissue, including miracidia, sporocysts, cercariae, schistosomula, juveniles, adult males, adult females and gonads (testes and ovaries)^31^; also, some of the stage/tissues are represented by more than one module (Fig. 2A) (please refer to “Materials and methods” section for details).

Evaluating to which of the lncRNAs/mRNAs co-expression modules the lncRNAs differentially expressed after 5-AzaC treatment belong to, can help the understanding of the gene expression programs altered by this epigenetic drug on *S. mansoni* females. When we looked at the modules to which the 552 lncRNAs upregulated after 5-AzaC exposure in females belong to, we observed enrichment in a male-related module (Fig. 2B). Out of the 552 lncRNAs upregulated after 5-AzaC treatment, 450 were assigned to any module, being the top three most represented enriched modules: adult males (yellow module, 114 lncRNAs or 20% of the lncRNAs, p value < 0.0001, hypergeometric test), gonads (brown module, 71 lncRNAs or 13% of the lncRNAs, p value < 0.001, hypergeometric test) and schistosomula (magenta module, 49 lncRNAs or 9% of the lncRNAs, p value < 0.01, hypergeometric test) (Fig. 2B).

Out of the 360 lncRNAs downregulated after 5-AzaC treatment, 296 were assigned to any module, being the three top most represented modules: adult males (turquoise module, 98 lncRNAs or 27% of the lncRNAs, p value < 0.0001, hypergeometric test), gonads (brown module, 29 lncRNAs or 8% of the lncRNAs, p value = 0.057, hypergeometric test) and adult females (pink module, 27 lncRNAs or 7% of the lncRNAs, p value < 0.01, hypergeometric test) (Fig. 2C). It is possible that 5-AzaC treatment in females switches the lncRNA transcriptional program to a pattern more similar to that shown by males, as it was shown for protein-coding genes in females treated with GSK343, an histone methyltransferase EZH2 inhibitor^34^, or in unpaired females, in which the gonads are not developed^35^. This effect can impact on stem cell activity and egg production by females, as previously shown by Geyer et al.^16^.

Similar patterns of module distribution of protein-coding genes in the Maciel et al.^31^ dataset (Fig. 2D) and of protein-coding genes differentially expressed after 5-AzaC treatment (Fig. 2E,F) were obtained in our re-analysis of the Geyer et al*.*^16^ dataset.

The list of lncRNAs and protein-coding genes differentially expressed after 5-AzaC treatment, as well as the modules to which they belong are given in Supplementary Table S4.

### Involvement of lncRNAs with the parasite reproductive biology

In order to evaluate if the lncRNAs differentially expressed after 5-AzaC treatment could be involved in *S. mansoni* reproductive biology, we checked if these lncRNAs are also differentially expressed in pairing-dependent conditions or in reproductive organs compared with whole worms. To do that, we cross compared the lncRNAs differentially expressed after 5-AzaC exposure in females with lncRNAs that we found to be differentially expressed in a re-analysis of the Lu et al. data^35^ (please refer to “Materials and methods” section for details) for lncRNAs differentially expressed between bisex (paired) females (bF) and single-sex females (sF), between bisex ovaries (bO) and single-sex ovaries (sO) and between bisex ovaries(bO) and bisex females (bF). We found that 60% of the lncRNAs downregulated after 5-AzaC exposure (216 out of 360 lncRNAs) are also present in at least one of these comparisons (Supplementary Fig. S2A,B, see overlap between the yellow oval and the other ovals). When the statistical significance of the overlaps between the lncRNAs downregulated after 5-AzaC exposure and each of the above comparisons was calculated, all of the overlaps were statistically significant, with all the p values obtained from the pairwise comparisons lower than 1.254e−10 (hypergeometric test).

In addition, 23% of the lncRNAs upregulated after 5-AzaC exposure (127 out of 552 lncRNAs) are also present in at least one of these comparisons (Supplementary Fig. S2C,D, see overlap between the yellow oval and the other ovals). When the statistical significance of the overlaps between the lncRNAs upregulated after 5-AzaC exposure and each of the comparisons was calculated, all of the overlaps were statistically significant (with all the p values obtained for the pairwise comparisons lower than 0.037, hypergeometric test), except for two pairwise comparisons: “lncRNAs upregulated after 5-AzaC versus bF > sF” (p value = 0.119) and “lncRNAs upregulated after 5-AzaC versus sF > bF” (p value = 0.181).

It has been shown that juvenile worms and schistosomula co-express transcripts that cluster into modules midnightblue and magenta, respectively^31^. These modules are among those with higher lncRNA/total transcripts ratio when compared with all modules: midnightblue is the second and magenta is the fourth module, out of 15 modules, with the highest lncRNAs/total transcript ratios. In midnightblue and magenta modules, lncRNAs correspond to 80% and 64% of all the transcripts, respectively^31^. As a high proportion of lncRNAs upregulated by 5-AzaC in *S. mansoni* females belongs to midnightblue and magenta modules (16.7% of all upregulated lncRNAs assigned to any module), we tested if 5-AzaC treatment would have any impact on schistosomula viability. We treated schistosomula with different concentrations of 5-AzaC and measured the viability at each day, along 5 days of treatment. No statistically significant reduction in schistosomula viability as measured by ATP levels was observed after 5-AzaC treatment at any of the concentrations and days tested (Supplementary Fig. S3A), with discrete phenotypic alterations observed only at day 5 post-treatment, at 245 µM, the highest concentration tested (Supplementary Fig. S3B). This is in agreement with observations that *S. mansoni* schistosomula possess lower detectable levels of 5-methylcytosine and of mRNAs encoding SmDnmt2 and SmMBD proteins involved with DNA methylation^15^, compared with other *S. mansoni* life-cycle stages, thus probably making schistosomula less susceptible to 5-AzaC treatment.

### LncRNAs differentially expressed upon 5-AzaC treatment have histone marks at their genomic ***loci***

The presence of histone marks at the TSSs of lncRNAs adds another layer of functionality evidence for lncRNAs, indicating regulation by epigenetic mechanisms related to chromatin structure^26,31^. Therefore, to check if the lncRNAs differentially expressed after 5-AzaC treatment would have histone marks at their TSSs, we cross compared the lncRNAs affected by 5-AzaC treatment with lncRNAs expressed in *S. mansoni* and reported by Maciel et al.^31^ as having at least one histone mark obtained by ChIP-Seq (H3K4me3, that is generally associated with active transcription or H3K27me3, associated to transcription repression) in non-treated *S. mansoni* cercariae, schistosomula or adults. As reported in that work^31^, 8599 out of 16,583 lncRNAs identified in different *S. mansoni* life-cycle stages and tissues have at least one histone modification mark within 1 kb from their TSS^31^. In addition, gene expression control by DNA/RNA methylation, affected by 5-AzaC, has been linked to histone modifications in eukaryotes^36,37^.

A total of 461 out of 912 lncRNA transcripts differentially expressed after 5-AzaC treatment have at least one histone modification mark within 1 kb from their TSS, being 274 upregulated lncRNAs (Supplementary Table S5) and 187 downregulated lncRNAs (Supplementary Table S6). This represents 50% of all the 912 lncRNAs differentially expressed after 5-AzaC treatment, which is statistically significant (p value < 0.05, hypergeometric test).

**Figure 3:**
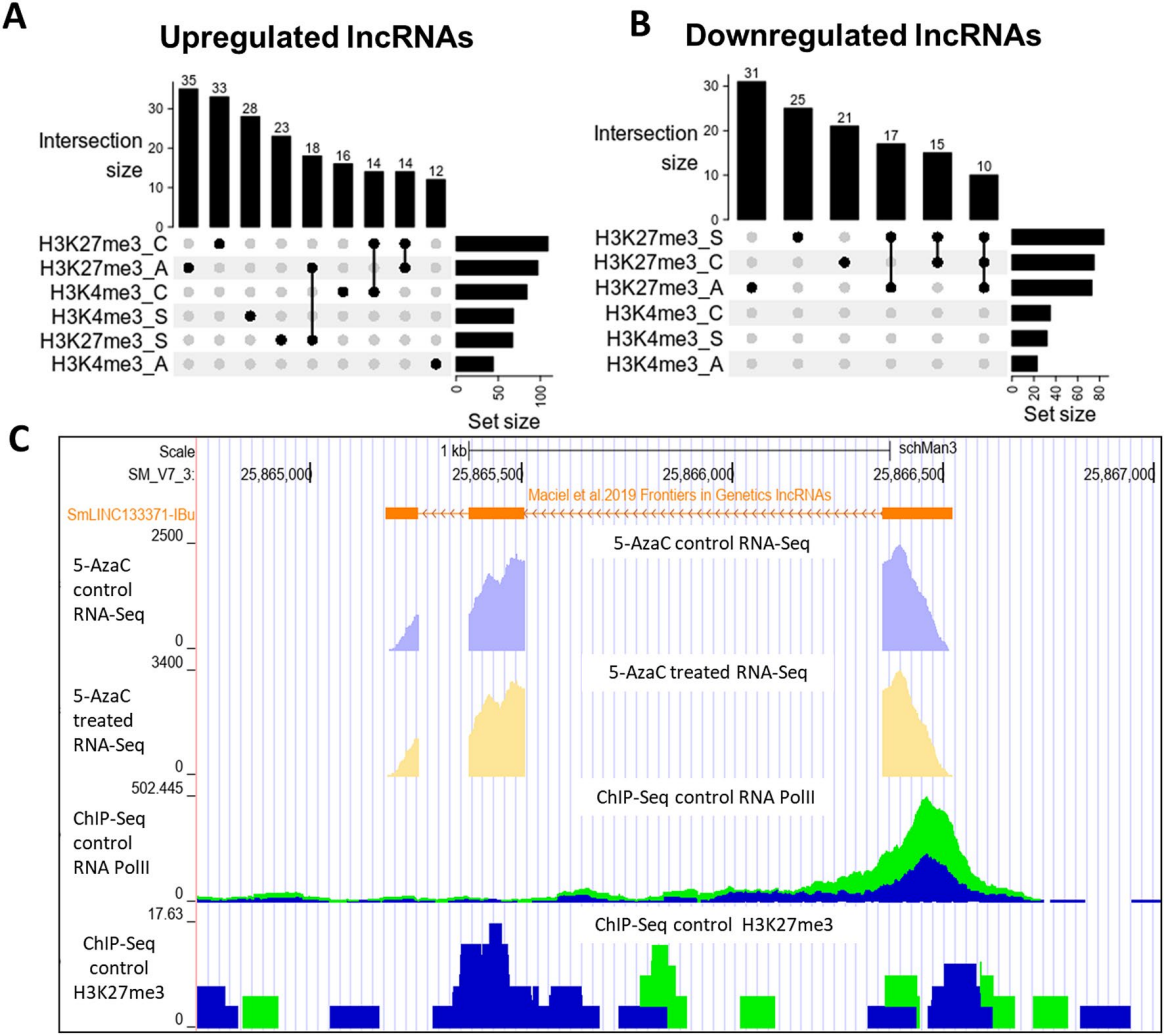
Hundreds of lncRNAs differentially expressed after 5-AzaC exposure in *S. mansoni* females have histone transcriptional activating or repressive marks at their TSSs. The UpSet intersection diagram shows the number of *S. mansoni* lncRNAs differentially expressed after 5-AzaC exposure (y-axis) that have been detected in each of the intersection sets, indicated by the connected points in the lower part of the plot, as having the H3K4me3 transcriptional activating marks and/or the H3K27me3 repressive marks within 1 kb (upstream or downstream) from their TSSs. Six histone mark datasets indicated at the bottom left were analyzed: H3K4me3_A in adults, H3K4me3_C in cercariae, H3K4me3_S in schistosomula, H3K27me3_A in adults, H3K27me3_S in schistosomula, and H3K27me3_C in cercariae, and each set size horizontal black bar represents the number of lncRNAs that contain the indicated histone mark at the indicated stage. The top enriched intersection sets are shown for the 5-AzaC upregulated (**A**) and downregulated (**B**) lncRNAs; all intersection sets and the lists of lncRNAs in each intersection set are shown in Supplementary Table S5, S6. (**C**) Snapshot of a *S. mansoni* genome browser image (www.schistosoma.usp.br), showing a region spanning 3 kb on chromosome 3, where the SmLINC133371-IBu is located. The orange track (top) represents lncRNAs from *S. mansoni* published by Maciel et al.^31^. Below the orange lncRNAs track, two other tracks show RNA-Seq data from control (light purple) or 5-AzaC treated *S. mansoni* females (light yellow). Below, two ChIP-Seq tracks are shown: RNA Polymerase II ChIP-Seq (ChIP-Seq Control RNA Pol II) and H3K27me3 histone mark ChIP-Seq (ChIP-Seq Control H3K27me3). The green and blue colours at the two ChIP-Seq tracks at the bottom represent each of two experimental biological replicates.

The most abundant mark found individually at the *loci* of the lncRNAs differentially expressed after 5-AzaC treatment was H3K27me3 in adults, for both upregulated lncRNAs (with 35 marks, Fig. 3A) and downregulated lncRNAs (with 31 marks, Fig. 3B). The second and third most present marks were, among the upregulated lncRNAs, H3K27me3 in cercariae and H3K4me3 in schistosomula, and among the downregulated lncRNAs H3K27me3 in schistosomula and H3K27me3 in cercariae.

In addition, when computed together with other marks, the most abundant mark found in the upregulated lncRNAs upon 5-AzaC treatment (Fig. 3A) was the transcriptional repressive mark, H3K27me3, with 18 lncRNAs presenting this mark in adults and schistosomula and other 14 lncRNAs presenting this mark in adults and cercariae simultaneously. For the downregulated lncRNAs upon 5-AzaC treatment (Fig. 3B), H3K27me3 was also the most abundant mark found when the marks were computed together, with 17 lncRNAs presenting this mark in adults and schistosomula and other 10 lncRNAs presenting this mark in adults, schistosomula and cercariae simultaneously.

In Fig. 3C, we show the *locus* on chromosome 3 of SmLINC133371-IBu (orange track), a lincRNA that has H3K27me3 histone mark ChIP-Seq peaks (“ChIP-Seq Control H3K27me3” track at the bottom of the image) in adults (blue/green tracks). This lincRNA is upregulated 1.5 × in females after 5-AzaC treatment (yellow track, “5-AzaC treated RNA-Seq”), belongs to the greenyellow module and also has RNA Polymerase II peaks (“ChIP-Seq Control RNAPol II”) at its *locus*.

### Validation of lncRNAs differential expression by RT-qPCR

We designed PCR primer pairs for a selected set of ten genes, including eight lincRNAs and two protein-coding genes, to validate their differential expression after 5-AzaC treatment. First, we treated adult worm couples with 5-AzaC at 491 µM for 48 h, extracted RNA from females and males separately and then performed RT-qPCR. As observed by Geyer et al.^15^, 5-AzaC was not lethal to adult worms even when they were treated with 5-AzaC at 491 µM, the limit of aqueous solubility. Here, we measured for the first time the amount of ATP in adult worms upon 5-AzaC exposure, as readout for worm viability. 5-AzaC exposure for 48 h did not alter significantly the ATP content of adult worms when compared with the controls (Supplementary Fig. S4A, p = 0.12). In addition, we observed a statistically significant 49% reduction in egg laying by adult worms treated with 5-AzaC at 491 µM (p = 0.02, Supplementary Fig. S4B). Eggs laid by adult worms treated with 5-AzaC show many phenotypic abnormalities, including lack of lateral spine on some eggs and eggs with smaller sizes (Supplementary Fig. S4C).

Quantitative real-time PCR (RT-qPCR) was then employed to validate results obtained by the RNA-Seq analysis. Two protein-coding genes were used as controls: Smp_151640 (*Insulin-like growth factor I*), which was 14.6 × upregulated in the RNA-Seq after 5-AzaC treatment (Supplementary Fig. S5A, left) and Smp_121390 (*Genome polyprotein*), which was downregulated 3.6 × in the RNA-Seq data (Supplementary Fig. S5B, left). In the RT-qPCR, both protein-coding genes were validated in females: Smp_151640 was upregulated 12.1 × (Supplementary Fig. S5A, right) and Smp_121390 was downregulated 2.5 × (Supplementary Fig. S5B, right) after 5-AzaC treatment. In addition, we also tested the expression of both Smp_151640 and Smp_121390 after 5-AzaC treatment in *S. mansoni* males. While Smp_151640 was found to be 51.4 × upregulated (Supplementary Fig. S6A), Smp_121390 was not differentially expressed after 5-AzaC in vitro treatment in males (Supplementary Fig. S6B).

We then tested by RT-qPCR in *S. mansoni* females and males a selected set of eight lincRNAs found to be differentially expressed in the female RNA-Seq dataset: SmLINC133371-IBu, SmLINC151825-IBu, SmLINC158444-IBu, SmLINC110084-IBu, SmLINC158969-IBu, SmLINC156349-IBu, SmLINC103888-IBu and SmLINC100882-IBu. These lincRNAs were selected because they show a wide range of expression levels in the RNA-Seq (TPM from 4 to 1635 in at least one of the conditions, control or 5-AzaC treated), because they have fold-changes higher than 1.5 × in the RNA-Seq dataset and because they all show only one isoform at their loci, except for SmLINC151825-IBu.

**Figure 4:**
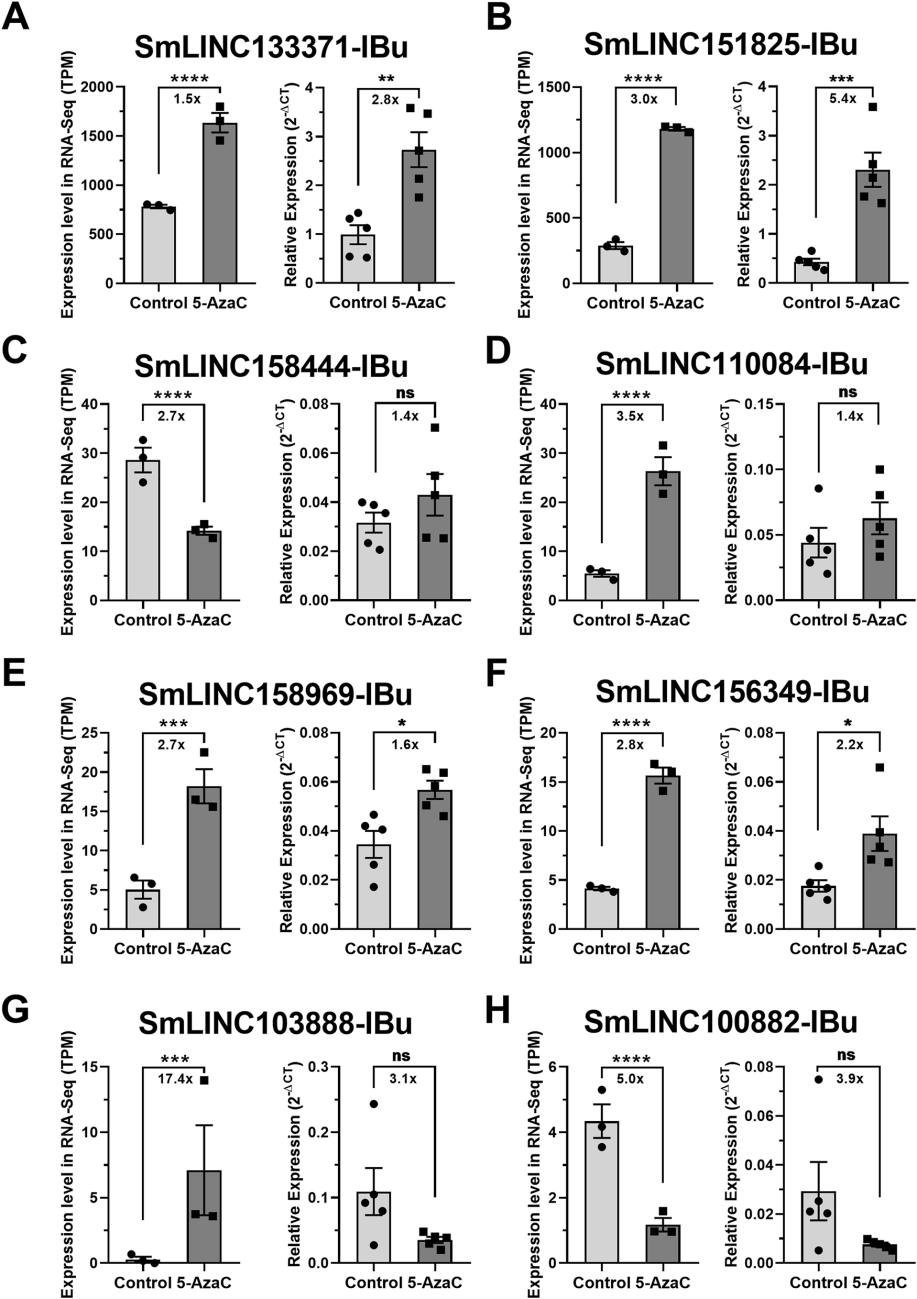
Expression profiles in *S. mansoni* females of selected lincRNAs differentially expressed after 5-AzaC treatment (491 µM). Eight lincRNAs were selected after re-analysis of RNA-Seq public datasets of 5-AzaC treated *S. mansoni* females^16^ for validation by RT-qPCR in females. For each of the eight selected lincRNAs, the expression profiles obtained with RNA-Seq are shown on the left as TPM (transcripts per million), whereas the RT-qPCR results are shown on the right: (**A**) SmLINC133371-IBu; (**B**) SmLINC151825-IBu; (**C**) SmLINC158444-IBu; (**D**) SmLINC110084-IBu; (**E**) SmLINC158969-IBu; (**F**) SmLINC156349-IBu; (**G**) SmLINC103888-IBu; (**H**) SmLINC100882-IBu. For the RNA-Seq data, three biological replicates were analyzed; the fold-changes and p values represented by asterisks that are shown in the brackets were obtained using DESeq2. For the RT-qPCR data, mean ± SEM from five biological replicates are shown, and Student unpaired two-sided t test was applied. *p < 0.05, **p < 0.01, ***p < 0.001, ****p < 0.0001; ns: not significant.

Four of these lincRNAs (SmLINC133371-IBu, SmLINC151825-IBu, SmLINC158969-IBu and SmLINC156349-IBu) were validated by RT-qPCR in females, confirming the RNA-Seq data; in our assays with *S. mansoni* females, they were upregulated 2.8 ×, 5.4 ×, 1.6 × and 2.2 ×, respectively (Fig. 4A,B,E,F). Additionally, four other lincRNAs tested were detected as expressed in the RT-qPCR assays; however, they were not differentially expressed after 5-AzaC treatment as predicted by the RNA-seq data (Fig. 4C,D,G,H). This indicates that there is variability of lncRNA expression and response to 5-AzaC exposure, probably related to the different parasite strains used in our RT-qPCR assays and in the RNA-Seq experiments from the literature^16^.

Considering the six genes in which the effect of 5-AzaC was validated in females by RT-qPCR (four lincRNAs and the two protein-coding genes), the extent of the effect measured by RT-qPCR mirrored the one obtained with RNA-Seq, as fold changes in expression were well correlated (Pearson correlation coefficient = 0.9334, p value = 0.0065, Supplementary Fig. S7).

**Figure 5:**
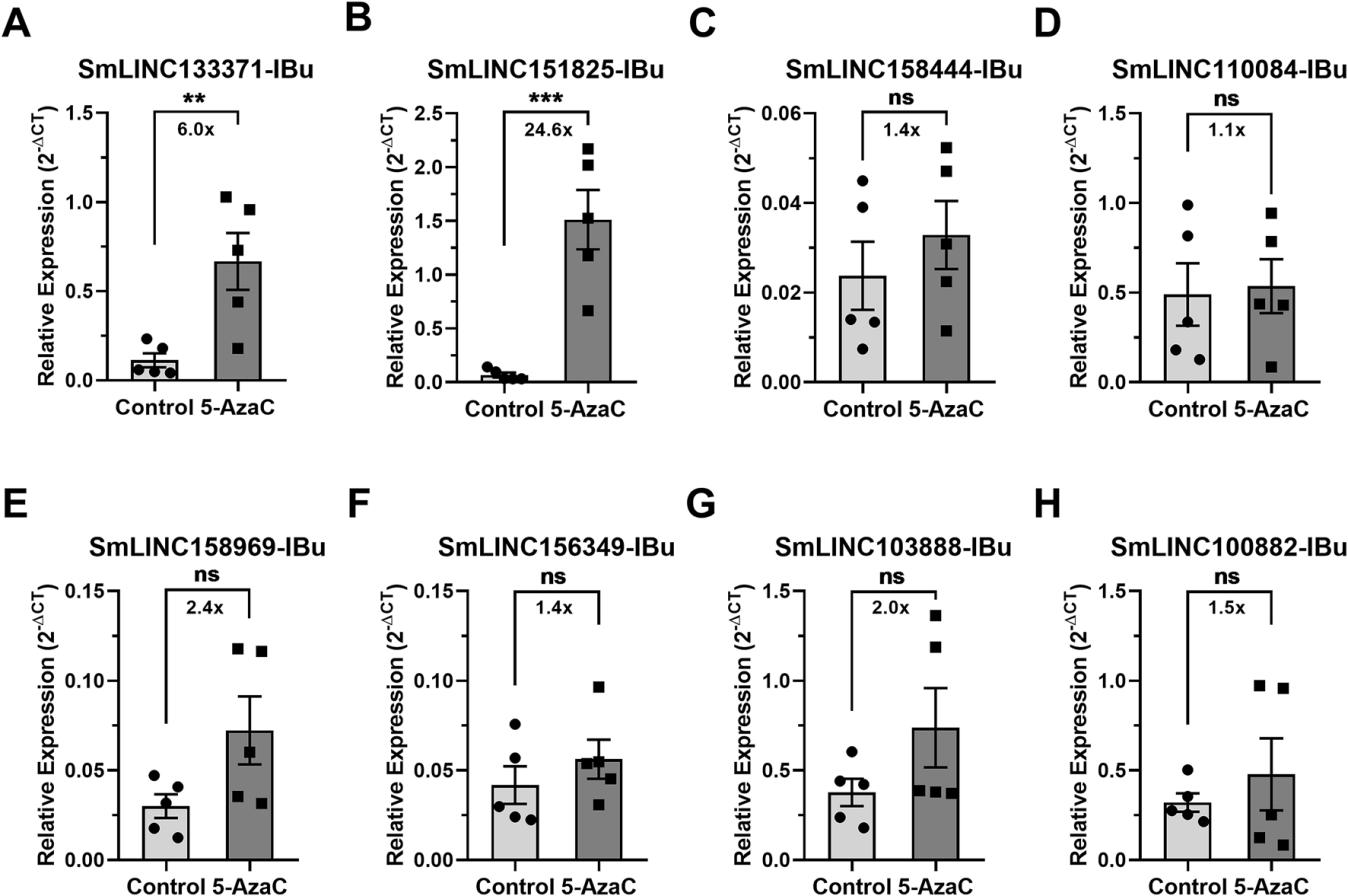
Expression profiles in *S. mansoni* males of selected lincRNAs differentially expressed after 5-AzaC treatment (491 µM). Eight lincRNAs were selected after re-analysis of RNA-Seq public datasets of 5-AzaC treated *S. mansoni* females^16^ for evaluation of differential expression by RT-qPCR in *S. mansoni* males. For each of the eight lincRNAs, the expression profiles in controls and in 5-AzaC treated *S. mansoni* males by RT-qPCR are shown: (**A**) SmLINC133371-IBu; (**B**) SmLINC151825-IBu; (**C**) SmLINC158444-IBu; (**D**) SmLINC110084-IBu; (**E**) SmLINC158969-IBu; (**F**) SmLINC156349-IBu; (**G**) SmLINC103888-IBu; (**H**) SmLINC100882-IBu. Mean ± SEM from five biological replicates are shown, and Student unpaired two-sided t test was applied; **p < 0.01, ***p < 0.001; *ns* not significant.

Interestingly, two out of the eight lincRNAs that were tested (SmLINC133371-IBu and SmLINC151825-IBu) were also upregulated in males treated with 5-AzaC (6.0 × and 24.6 ×, respectively, Fig. 5A,B), indicating that these lincRNAs share similar regulatory mechanisms in both sexes. Expression of the other six tested lincRNAs was not significantly affected by 5-AzaC treatment of males (Fig. 5C–H).

### LncRNAs modulated by 5-AzaC are differentially expressed along ***S. mansoni*** life-cycle stages

To evaluate if the lncRNAs differentially expressed after 5-AzaC treatment tested by RT-qPCR here are also expressed in other *S. mansoni* life-cycle stages or tissues, we re-analyzed data from public RNA-Seq libraries from different *S. mansoni* life-cycle stages and tissues (Supplementary Table S7) to look for the expression patterns of the eight selected lincRNAs. First, we evaluated the stage-specificity of the different RNA-Seq datasets that we used for this re-analysis by confirming that five protein-coding genes previously described as stage markers^38,39^ were indeed more highly expressed at the predicted stages in our analysis (Supplementary Fig. S8). In addition, PCA analysis (Supplementary Fig. S9) shows that biological replicates of the same sample grouped according to the life-cycle stages and tissues, confirming the clustering of samples in expected segregating groups.

**Figure 6:**
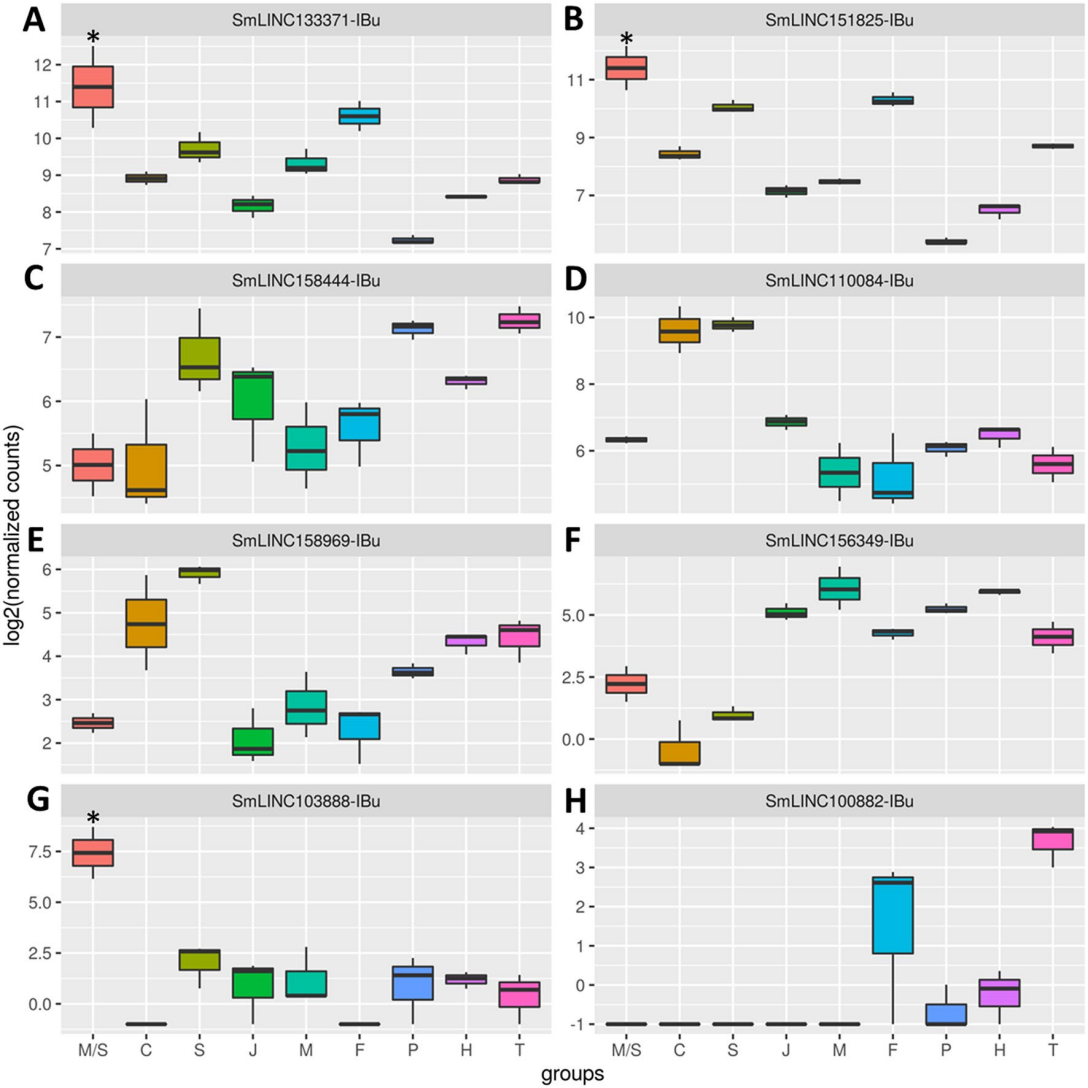
RNA-seq expression profiles at different *S. mansoni* stages of selected lincRNAs differentially expressed after 5-AzaC treatment (491 µM). The expression levels (shown as log2 of normalized counts) of the eight lincRNAs whose gene IDs are indicated at the top of each panel are shown. These lincRNAs were selected after re-analysis of RNA-Seq public datasets of 5-AzaC treated *S. mansoni* females^16^. The y-axis shows the expression level for each lincRNA in the RNA-seq assays (log2 of normalized counts) as determined at the stage indicated in the x-axis as follows: miracidia/sporocysts (M/S), cercariae (C), schistosomula (S), juveniles (J), adult males (M), adult females (F), posterior somatic tissues (P), heads (H) and tails (T). (**A**) SmLINC133371-IBu; (**B**) SmLINC151825-IBu; (**C**) SmLINC158444-IBu; (**D**) SmLINC110084-IBu; (**E**) SmLINC158969-IBu; (**F**) SmLINC156349-IBu; (**G**) SmLINC103888-IBu; (**H**) SmLINC100882-IBu. Only transcripts that were upregulated in one stage/tissue when compared with all others were considered as significantly more expressed in that stage/tissue and are marked with an asterisk. *p value < 0.05.

We then looked at the expression levels along *S. mansoni* life-cycle stages and tissues of the eight lincRNAs tested by RT-qPCR and observed a heterogeneous expression pattern distribution (Fig. 6). Expression of SmLINC133371-IBu (Fig. 6A), SmLINC151825-IBu (Fig. 6B) and SmLINC103888-IBu (Fig. 6G) is higher in miracidia and sporocysts stages, with SmLINC133371-IBu and SmLINC151825-IBu being also highly expressed in adult females and schistosomula (Fig. 6A,B). Whereas SmLIN158444-IBu shows higher expression in the posterior adult somatic tissues and tails (Fig. 6C), SmLINC110084-IBu has higher expression in schistosomula and cercariae (Fig. 6D). While SmLINC156349-IBu (Fig. 6E) and SmLINC158969-IBu (Fig. 6F) show broad expression in all the stages, SmLINC100882-IBu (Fig. 6H) is highly expressed in female adult worms and tails. These results show that most of the tested lncRNAs (except SmLINC103888-IBu and SmLINC100882-IBu) are not stage-specific and may play roles in other *S. mansoni* life-cycle stages.

## Discussion

Here, we have shown that long non-coding RNAs levels can be modulated in *S. mansoni* by 5-AzaC, a DNA methyltransferase inhibitor that is currently used to treat myelodysplastic syndrome and acute myeloid leukemia in humans^12,40^. Hundreds of the lncRNAs differentially expressed after 5-AzaC exposure in *S. mansoni* females belong to co-expression modules related to male metabolism, have histone marks at their genomic *loci* and are also differentially expressed in unpaired compared with paired *S. mansoni* females and ovaries. While short RNAs (especially miRNAs) have been more explored in various helminths^41–44^, lncRNAs have received little attention, being identified by transcriptomic approaches only in a few helminths other than *S. mansoni*^22–24^ or studied in a limited number of free-living nematodes^45,46^. In addition, unlike miRNAs^47–52^, the mechanisms of regulation of lncRNAs are largely unknown in parasites and, to our knowledge, this is the first report of modulation of lncRNAs levels by an epigenetic drug in any helminth.

In the past few years, human lncRNAs have been proposed as drug targets in many diseases, especially in cancer and neurological syndromes^21,53–55^. In parasitic diseases, there is a clear need to develop new and inexpensive drugs, especially with the emerging reports of drug resistance^6,56–58^. We believe that it is time to consider lncRNAs as possible drug targets also in parasitic diseases, especially because they show lower conservation in their primary sequences between species than protein-coding genes^17,19,59,60^, which in principle would reduce side effects in therapeutic strategies.

The choice of lncRNAs to be further validated as drug targets will rest on the appropriate selection of lncRNA candidates. This selection should be guided by functional characterization of the lncRNA as well as by the demonstration of the lncRNA relevance to the parasite biology. Here, we found that 38% of the lncRNAs differentially expressed after 5-AzaC treatment in *S. mansoni* females (343 out of 912 lncRNAs) are also differentially expressed between paired and unpaired females or ovaries^35^, whilst 24% of them (221 out of 912 lncRNAs) belong to co-expression modules related to “gonads”^31^, indicating an important involvement of lncRNAs on parasite sexual maturation and reproductive biology. In addition, 50% of the lncRNAs differentially expressed after 5-AzaC treatment in *S. mansoni* females (461 out of 912 lncRNAs) have at least one histone mark at their TSSs previously detected at *S. mansoni* life-cycle stages^31^. These lncRNAs with evidence of chromatin marks at their genomic loci could be prioritized in further functional assays to elucidate their relevance, roles and mechanisms of action in *S. mansoni* biology. Technologies for lncRNAs targeting should be considered in these studies, including cell and tissue localization, silencing by CRISPR or antisense oligonucleotides methods in vitro and in vivo, and discovery of lncRNA partners (DNA, RNA or proteins)^61–63^.

The expression patterns along life-cycle stages may also be criteria for the selection of lncRNAs to be tested in functional assays. Some of the lncRNAs tested by RT-qPCR here in *S. mansoni* female and male adult worms are also expressed at high levels (TPM > 100) in other life-cycle stages, including SmLINC133371-IBu and SmLINC151825-IBu with high expression levels in miracidia, sporocysts and schistosomula. In addition, SmLINC158444-IBu and SmLINC110084-IBu are highly expressed in posterior somatic tissues and schistosomula, respectively. All these lncRNAs, except SmLINC100882-IBu are expressed in schistosomula, another life-cycle stage of interest regarding drug targeting, as praziquantel has no efficacy against schistosomula^64^.

Additionally, many lncRNAs have been associated with drug resistance in human cancers^65–67^. Here, by measuring ATP levels, we confirm that 5-AzaC treatment has no effect on the viability of *S. mansoni* adult worms, as previously shown^15,16^. Moreover, we show that *S. mansoni* schistosomula viability is also not affect by 5-AzaC. It is unclear why the parasites’ viability is not affected by 5-AzaC, but since schistosomes show nucleoside auxotrophy^68^, precise regulation of nucleoside analogs uptake may control their toxicity. It is also possible that the lncRNAs differentially expressed after 5-AzaC exposure may be involved in a 5-AzaC drug resistance mechanism, as shown for human cancer-related lncRNAs such as *HOTAIR* and *XIST*^69–71^.

Understanding the mechanisms of lncRNA expression regulation may help the selection of lncRNAs for the development of new therapeutic strategies in the future. These mechanisms, which include epigenetic regulation by histone modification^72–74^ at lncRNA genomic *loci* and DNA/RNA methylation already described in human lncRNAs^75–78^ are, however, less understood than those of protein-coding genes^79,80^. It is now clear that epigenetic processes play important roles on schistosomes^81–83^. In fact, epigenetic mechanisms participate in schistosome phenotypic plasticity^84,85^, in egg production and adult worm viability^34,86,87^ as well as in schistosomula survival^88–92^. DNA methylation, one of the most studied epigenetic mechanisms, has been detected in *S. mansoni*^15^, although the significance of DNA cytosine methylation (5mC) in this parasite has been somewhat controversial^93–95^. Here, we observed that 912 lncRNAs are differentially expressed after 5-AzaC exposure in *S. mansoni* females, all of them expressed at an average TPM > 0.1 in control or 5-AzaC treated samples. As previous analysis identified 9229 lncRNAs expressed in females (TPM > 0.1) out of all 16,583 detected at any *S. mansoni* life-cycle stage^31^, we estimate that 10% of all lncRNAs expressed in females are differentially expressed upon 5-AzaC exposure.

Although the presence of DNA methylation in many invertebrates has been already reported^96,97^, previous work was unable to detect functional roles of DNA methylation in invertebrates^98^. 5-AzaC is an inhibitor of DNA methyltransferase that has been shown in *S. mansoni* to inhibit female specific biological processes including egg production, egg maturation and normal ovarian development^15,16^, phenotypic effects confirmed in our treatments. These effects are achieved by modifications of adult female transcription and translation, with 81% inhibition in de novo protein synthesis in female schistosomes^16^. As 5-AzaC incorporates preferentially into RNAs, with only 20% being incorporated into DNA^99^, it is more likely that 5-AzaC interferes preferentially in lncRNA stability through lncRNA methylation impediment than through promoter DNA methylation. In fact, many lncRNAs were shown to be regulated by RNA methylation in humans and in *Arabidopsis*^100–102^, although some human lncRNAs have been also identified as regulated by DNA methylation at their promoter regions^32,33,103^.

5-AzaC also affects *S. mansoni* females’ stem cells, leading to a 95% reduction in the number of proliferating stem cells^16^. Remarkably, lncRNAs actively participate in human stem cell pluripotency, maintenance and differentiation^104^. Thus, it is possible that some of the lncRNAs found here as modulated by 5-AzaC play important roles in parasite stem cells. Further analyses of lncRNA expression in spatially-distinct *S. mansoni* female stem cell populations under 5-AzaC exposure, including vitelline S1 stem cells which are vital for egg production, may uncover lncRNA functional roles on stem cell maintenance. Alternatively, 5-AzaC may modulate lncRNA expression levels by exerting pleiotropic effects similar to those reported in human cell lines such as suppressing lipid metabolism^105^, inhibition of pathways that regulate DNA synthesis/repair^106^ or de-repression of retroviral expression^107^. Future studies aiming to elucidate the precise mechanism of action of 5-AzaC in lncRNA regulation^108,109^ could offer starting points for lncRNA targeting and manipulation in *S. mansoni*.

In summary, this study adds another layer on the understanding of the effects of 5-AzaC in *S. mansoni* and sheds light on the relevance of looking at lncRNA regulation in response to drug treatment in parasites. Although the use of 5-AzaC against schistosomiasis is unlikely as its effects are not parasite selective, the lncRNAs affected by 5-AzaC identified here, together with downstream pathways already described as affected by 5-AzaC, could represent new targets for the development of alternative chemotherapeutic strategies against schistosomiasis.

## Material and methods

### Analysis of 5-AzaC RNA-Seq data

Public RNA-Seq data from Geyer et al.^16^ for *S. mansoni* females were downloaded from the SRA-NCBI database (project number PRJNA428470; controls #SRR6490481, #SRR6490482 and #SRR6490483; treated with 491 µM 5-AzaC #SRR6490480, #SRR6490484 and #SRR6490485). Adapters and bad quality reads were filtered out using fastp v. 0.19.5 with default parameters^110^. For transcripts expression quantitation the genome sequence v.7, and a GTF file containing the protein-coding transcriptome v 7.1 were downloaded from the WormBase ParaSite resource (version WBPS14)^111^. The latter was merged with the lncRNA transcriptome sequences identified by Maciel et al.^31^ and the resulting GTF, which is available at http://schistosoma.usp.br/, was used as the reference. The filtered RNA-Seq reads were aligned with STAR v 2.7^112^ and quantified with RSEM v 1.3.1^113^, both using default parameters, and with the RSEM “estimate-rspd parameter on” option. Transcripts with counts lower than 10 were removed and differential expression analysis was performed using DESeq2 package^114^ v. 1.24.0 with an FDR threshold of 0.05. The Volcano plot shows the − log10(p value) vs log2(fold-change) for the lncRNAs obtained in the DESeq2 analysis, using EnhancedVolcano (R package version 1.6.0), available at https://github.com/kevinblighe/EnhancedVolcano. To look for the expression patterns of lincRNAs at different *S. mansoni* life-cycle stages and tissues we re-analyzed data from the public RNA-Seq libraries indicated in Supplementary Table S7, using the same pipeline described above. Pairwise differential expression analysis was performed between each two stages and/or tissues using DESeq2 with an FDR threshold of 0.05. Only transcripts that were upregulated in one stage/tissue when compared with all others, were considered as significantly more expressed in that stage/tissue.

PCA plot was obtained after normalization using the vst function followed by the plotPCA function from DESeq2.

### Analysis of the features of lncRNAs differentially expressed after 5-AzaC treatment

The lncRNAs differentially expressed in *S. mansoni* females after 5-AzaC treatment were compared with the lists of lncRNAs identified in four different datasets, namely: (1) lncRNAs belonging to one of the 15 weighted gene co-expression network analyses (WGCNA) modules previously published by Maciel et al.^31^ to check to which modules the lncRNAs differentially expressed after 5-AzaC treatment belong to. In Maciel et al.^31^, 90 libraries from *S. mansoni* miracidia, sporocysts, schistosomula, cercariae, and gonads (testes and ovaries) were analyzed using the unsupervised WGCNA^115^ co-expression analysis approach and 15 different WGCNA modules were obtained^31^, each of them representing one cluster of highly interconnected genes that are more expressed in a given stage/tissue. That analysis^31^ resulted in the identification of two modules representing miracidia/sporocysts (black and purple), two modules representing juveniles (blue and midnight blue), two modules representing adult males (turquoise and yellow) and four modules representing gonads (brown, green, greenyellow and salmon). Regarding the other five modules, each of them represents only one stage/tissue: cyan (ovaries), magenta (schistosomula), pink (adult females), red (testes) and tan (cercariae); (2) lncRNAs differentially expressed between bisex females (paired, bF) and single-sex females (unpaired, sF) and between bisex ovaries (paired, bO) and single-sex ovaries (unpaired, sO) and whole worms, which we determined by a re-analysis of the transcriptomes previously obtained by Lu et al.^35^ (see below); (3) lncRNAs previously published by Maciel et al.^31^ as having at least one histone mark (H3K4me3 or H3K27me3) at their TSSs, to check for the presence of histone marks at the TSS of lncRNAs differentially expressed after 5-AzaC treatment; (4) the expression patterns of lncRNAs along *S. mansoni* life-cycle stages, previously published by Maciel et al.^31^.

Data from Lu et al.^35^ were obtained from SRA (Project number PRJEB14695). In that work, Lu et al.^35^ performed RNA-Seq in bisex (paired) females (bF), single-sex females (sF), bisex (paired) males (bM), single-sex males (sM), bisex ovaries (bO), single-sex ovaries (sO), bisex testes (bT) and single-sex testes (sT), but only the protein-coding genes were analyzed. Here, a re-analysis of Lu et al.^35^ raw data to detect lncRNAs was performed using the same genome, annotation files and bioinformatics tools and parameters that were used to analyze the data from Geyer et al.^16^, as described above. The Venn diagram tool at http://jvenn.toulouse.inra.fr/app/index.html was used to compare the lists of lncRNAs detected as differentially expressed in the present study and in the gonad-specific and pairing-dependent study^35^.

### Parasite materials

All parasite materials were from a BH isolate of *S. mansoni* maintained by passage through golden hamster (*Mesocricetus auratus*) and *Biomphalaria glabrata* snails. Cercariae were collected from snails infected with 10 miracidia each. Thirty-five days after infection, the snails were placed in the dark in water and then illuminated for 2 h to induce shedding. Schistosomula were obtained by mechanical transformation of cercariae and separation of their bodies as previously described^116^, with some modifications. Briefly, cercariae were collected as described above and then suspended in 15 ml of M169 medium (Vitrocell, cat number 00464) containing penicillin/streptomycin, amphotericin (Vitrocell, cat number 00148). Mechanical transformation was performed by passing the cercariae 10 times through a 23G needle. To separate schistosomula from the tails, the tail-rich supernatant was decanted and the sedimented bodies resuspended in a further 7 ml of M169 medium. The procedure was repeated until less than 1% of the tails remained. The newly transformed schistosomula were maintained for 72 h in M169 medium (Vitrocell, cat number 00464) supplemented with penicillin/streptomycin, amphotericin, gentamicin (Vitrocell, cat number 00148), 2% fetal bovine serum, 1 μM serotonin, 0.5 μM hypoxanthine, 1 μM hydrocortisone, and 0.2 μM triiodothyronine at 37 °C and 5% CO_2_. Schistosomula cultivated for 72 h were used for 5-AzaC exposure. Adult *S. mansoni* worms were recovered by perfusion of golden hamsters that had been infected 7 weeks before with 250 cercariae, as previously described^116,117^. After perfusion, the adult worm pairs were kept for 3 h at 37 °C and 5% CO_2_ in DMEM (Gibco, catalogue number 11995-065-500) supplemented with 10% fetal bovine serum (FBS) (Vitrocell) and 100 mg/ml penicillin/streptomycin (Vitrocell). After 3 h of incubation, the adult worm pairs were used for 5-AzaC treatment.

### Parasite treatment with 5-AzaC

*Schistosoma mansoni* schistosomula and adult worms were treated with different final concentrations of 5-AzaC (Sigma, A2385) in culture medium specific to each stage: adult worms were treated with 5-AzaC at 491 μM (same treatment as in Geyer et al.^16^) and schistosomula were treated with 5-AzaC from 245 to 7.7 μM, as indicated in the Results. Adult male and female schistosome couples were cultivated in the presence (or absence) of 5-AzaC according to the methodology described in Geyer et al.^15^. 5-AzaC was added to 30 worm pairs for each of five biological replicates, while additional five replicates, lacking 5-AzaC, were included as controls. The schistosome cultures were incubated at 37 °C for 48 h in a humidified atmosphere with a 70% media exchange performed after 24 h. After 48 h, eggs were counted and schistosome worms were collected, washed three times with PBS and stored in RNA*later* (Ambion) until RNA extraction. Before the extraction of RNA from males or females, adult worm pairs were manually separated in RNA*later* (Ambion) using tweezers. Adult worm couple viability was evaluated 2 days post culture initiation using 9 worm pairs that were cultivated in 5 ml medium in 6 well tissue culture plates (n = 5 biological replicates; 5-AzaC at 491 μM treated or control worms).

Newly transformed schistosomula (NTS) were maintained in culture^34^ for 72 h and then treatment with 5-AzaC was initiated. Schistosomula viability was measured after 24, 48, 72, 96 and 120 h of treatment (n = 2 biological replicates).

### Viability assay

The viability of *S. mansoni* schistosomula and adult worms after treatment with 5-AzaC was determined by a cytotoxicity assay based on the CellTiter-Glo Luminescent Cell Viability Assay (G7570, Promega)^34,118^. The assay determines the amount of ATP present in freshly lysed adults or in intact schistosomula; the assay signals the presence of metabolically active cells.

### RNA extraction, quantification, and quality assessment

RNA extraction, quantification, and quality assessment were performed according to Maciel et al.^31^. Male or female adult worms were first disrupted in Qiagen RLT buffer using glass potters and pestles. RNAs from males or females were then extracted and purified using the Qiagen RNeasy Mini Kit (Cat number 74104), according to the manufacturer’s instructions, except for the DNase I treatment: the amount of DNase I was doubled, and the time of treatment was increased to 45 min.

The integrity of all RNAs was verified using the Agilent RNA 6000 Pico Kit (5067-1513 Agilent Technologies) in a 2100 Bioanalyzer Instrument (Agilent Technologies) and quantified using the Qubit RNA HS Assay Kit (Q32852, Thermo Fisher Scientifc). Purity was assessed by 260/280 nm and 260/230 nm ratios using Nanodrop (Thermo Fisher Scientific). Five biological replicates were assessed for 5-AzaC treated or control males or females.

### Reverse transcription and quantitative PCR (RT-qPCR) assays

The reverse transcription (RT) reactions were performed with 200 ng total RNA of each control and 5-AzaC treated female samples and with 30 ng total RNA of each control and 5-AzaC treated male samples. For the RT reactions, the SuperScript IV FirstStrand Synthesis System (18091050; Life Technologies) and random hexamer primers were used in a 20 μl final volume. The obtained complementary DNAs (cDNAs) were diluted four times in DEPC water, and quantitative PCR was performed using 2.5 μl of each diluted cDNA in a total volume of 10 μl containing 1 × LightCycler 480 SYBR Green I Master Mix (04707516001, Roche Diagnostics) and 800 nM of each primer in a LightCycler 480 System (Roche Diagnostics). Primers for selected transcripts (Supplementary Table S8) were designed using the Primer 3 online tool, and each RT-qPCR was run in three technical replicates. The results were analyzed by comparative Ct method^119^. Ct values are shown in Supplementary Table S9. Real-time qPCR data were normalized in relation to the level of expression of two reference genes previously used in the literature, namely Smp_900000^120–122^ and Smp_123610^117^.

### Statistical analyses

Two-tailed unpaired t test was used for pairwise comparisons, and GraphPad Prism software was used to perform the analyses (version 8.0). Hypergeometric test was used for enrichment calculations, using the online https://stattrek.com/online-calculator/hypergeometric.aspx tool. Quantification of data are represented as mean ± SEM and p value thresholds were * < 0.05, ** < 0.01, *** < 0.001 and **** < 0.0001.

### Ethics statement

The experimental protocols were in accordance with the Ethical Principles in Animal Research adopted by the Brazilian College of Animal Experimentation (COBEA) and the protocol/experiments have been approved by the Ethics Committee for Animal Experimentation of Instituto Butantan (CEUAIB n˚ 1777050816).

## Supplementary Information

Supplementary Figures.

Supplementary Tables.

## Data Availability

All data generated or analyzed during this study are included in this published article (and its Supplementary Information files).
