# Supplementary material for: Long non-coding RNA levels can be modulated by 5-azacytidine in Schistosoma mansoni
Source: Sci Rep. 2020 Dec 9;10:21565. doi: 10.1038/s41598-020-78669-5 (PMC7725772; doi:10.1038/s41598-020-78669-5)
Supplement: Supplementary file 1 — Supplementary Figures. [file 41598_2020_78669_MOESM1_ESM.pdf]

Long non-coding RNA levels can be modulated by 5-Azacytidine in *Schistosoma mansoni*

Murilo S. Amaral[1], Lucas F. Maciel[1], Gilbert O. Silveira[1,2], Giovanna G. O. Olberg[1], João V. P. Leite[1]; Lucas K. Imamura[1], Adriana S. A. Pereira[1,2], Patricia A. Miyasato[1], Eliana Nakano[1], Sergio Verjovski-Almeida[1,2*]

[1]Laboratório de Parasitologia, Instituto Butantan, São Paulo, Brazil

[2]Departamento de Bioquímica, Instituto de Química, Universidade de São Paulo, São Paulo, Brazil

Supplementary Figures S1 to S9 ……… pages 2 to 11

Supplementary References …………….. page 12

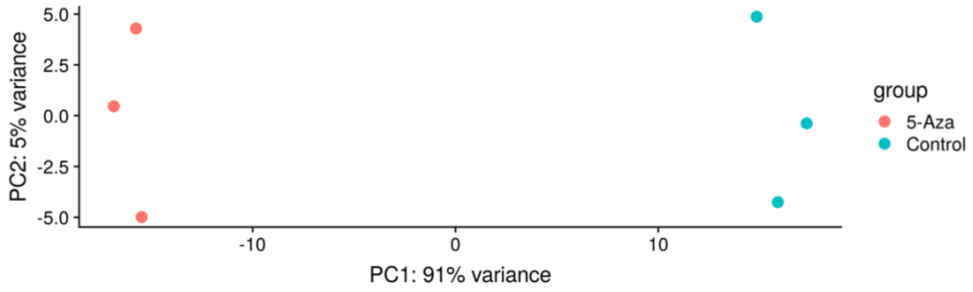

Supplementary Figure S1: Clustering of RNA-Seq biological replicates assessed by principal component analysis (PCA). RNA-Seq data from Geyer *et al*., 2018 [1] were re-analyzed using the *S. mansoni* genome PRJEA36577 (v.7) retrieved from WormBase and the recently published transcriptome that includes long non-coding RNAs [2] as reference. PCA plot was obtained after normalization using the vst function followed by the plotPCA function from DESeq2. Both control and 5-AzaC treated *S. mansoni* female samples are represented by three biological replicates each (n = 3), which are separated by their first two principal components. The control samples are represented by blue dots and the 5-AzaC treated samples by red dots.

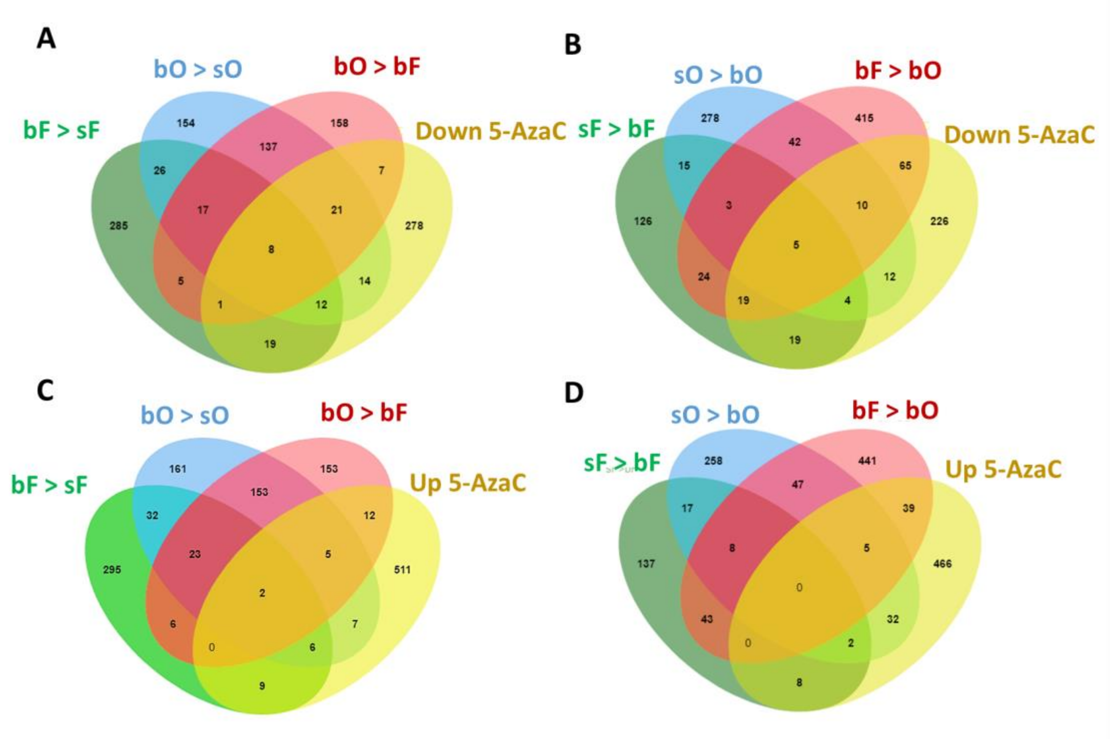

Supplementary Figure S2: Venn diagram representing the number of lncRNAs differentially expressed in different conditions of female pairing compared with females under 5-AzaC exposure. Re-analysis of RNA-seq public data from Lu *et al*., 2016 [3], focusing on lncRNAs mapping and quantification, followed by comparison with lncRNAs differentially expressed in bisex females after 5-AzaC exposure, as determined by re-analysis of data from Geyer *et al*., 2018 [1]. **A.** lncRNAs downregulated in bisex females after 5-AzaC exposure are compared with lncRNAs differentially expressed between the following conditions: lncRNAs enriched in bisex (paired) females compared with single-sex (unpaired) females (bF>sF); lncRNAs enriched in ovaries from bisex (paired) females compared with ovaries from single-sex (unpaired) females (bO>sO); lncRNAs enriched in ovaries from bisex (paired) females compared with bisex (paired) females (bO>bF). **B.** lncRNAs downregulated in bisex females after 5-AzaC exposure are compared with lncRNAs differentially expressed between the following conditions: lncRNAs enriched in single-sex (unpaired) females compared with bisex (paired) females (sF>bF); lncRNAs enriched in ovaries from single-sex (unpaired) females compared with ovaries from bisex (paired) females (sO>bO); lncRNAs enriched in bisex (paired) females compared with ovaries from bisex (paired) females (bF>bO). **C.** lncRNAs upregulated in bisex females after 5-AzaC exposure are compared with lncRNAs differentially expressed between the following conditions: lncRNAs enriched in bisex (paired) females compared with single-sex

(unpaired) females (bF>sF); lncRNAs enriched in ovaries from bisex (paired) females compared with ovaries from single-sex (unpaired) females (bO>sO); lncRNAs enriched in ovaries from bisex (paired) females compared with bisex (paired) females (bO>bF). **D.** lncRNAs upregulated in bisex females after 5-AzaC exposure are compared with lncRNAs differentially expressed between the following conditions: lncRNAs enriched in single-sex (unpaired) females compared with bisex (paired) females (sF>bF); lncRNAs enriched in ovaries from single-sex (unpaired) females compared with ovaries from bisex (paired) females (sO>bO); lncRNAs enriched in bisex (paired) females compared with ovaries from bisex (paired) females (bF>bO). Samples are labeled as bF: bisex (paired) females; sF: single-sex (unpaired) females; bO: bisex (paired) ovaries; sO: single-sex (unpaired) ovaries, according to Lu *et al*., 2016 [3].

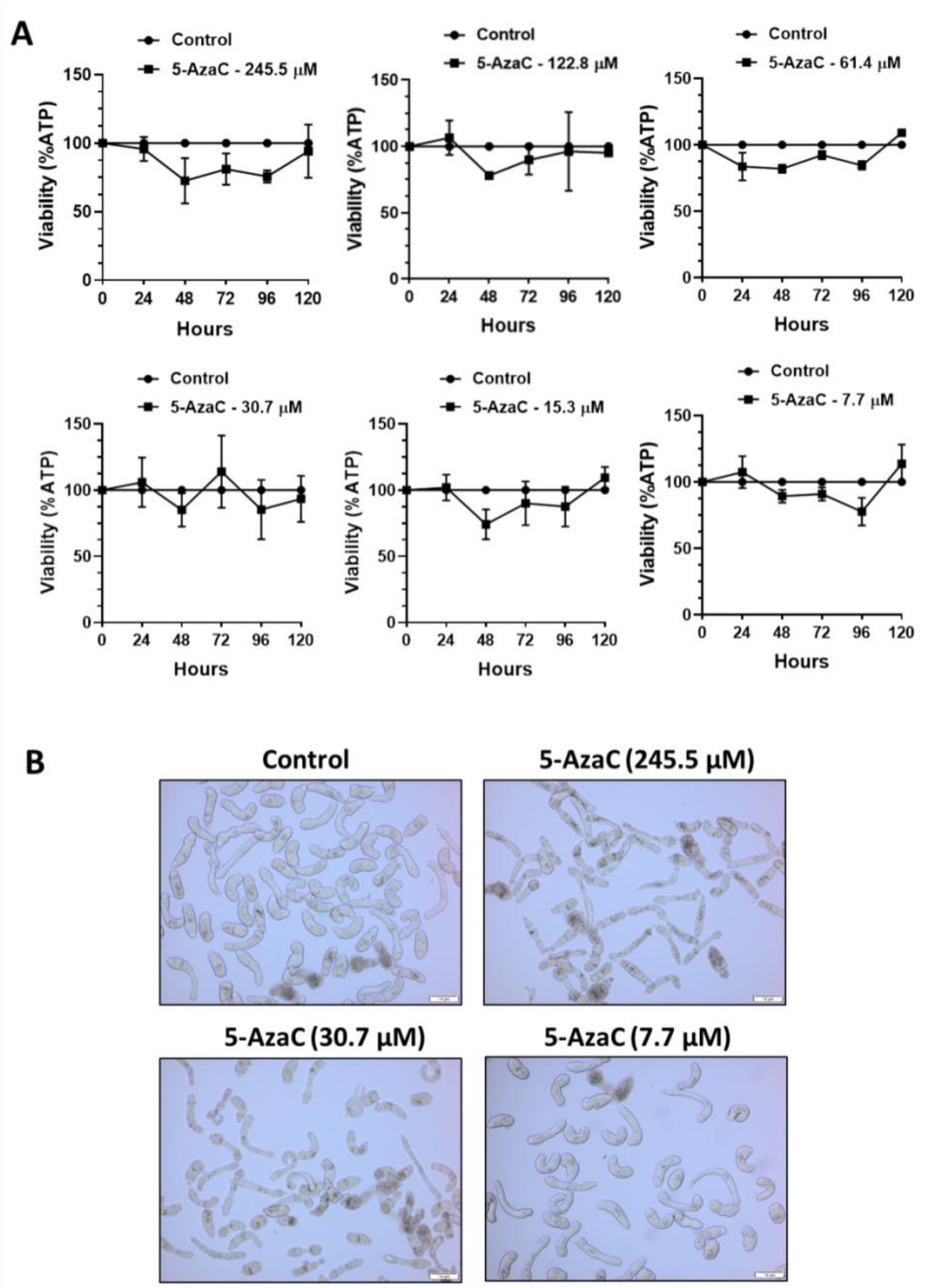

Supplementary Figure S3: Evaluation of *S. mansoni* schistosomula viability after 5-AzaC treatment at different concentrations and incubation times. (A) ATP quantitation using a luminescent assay to assess schistosomula survival under 5-AzaC exposure. *S. mansoni* schistosomula (100-110/well) were incubated with the indicated concentrations of 5-AzaC or with medium only (control) for 24, 48, 72, 96 and 120 h. Viability was expressed as % luminescence values relative to the control (medium only). Mean ± SEM from two replicate experiments. Two-way ANOVA was applied, and no statistically significant difference was found in any of the comparisons. **(B)** Light microscopy of schistosomula incubated with the indicated concentrations of 5-AzaC or with medium only (control) for 120 h. Scale bar: 100 µm.

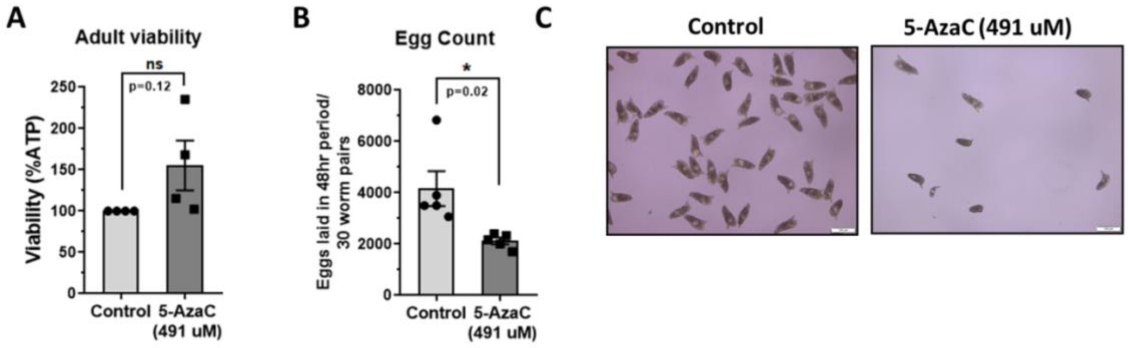

Supplementary Figure S4: Effect of 5-AzaC treatment on adult *S. mansoni* viability and egg laying. A. 5-AzaC does not affect adult *S. mansoni* viability. Nine worm pairs were cultivated in the presence (n = 5) or absence (n = 5) of 491 µM 5-AzaC for 48 h. Worms were collected and ATP levels were measured in control and treated worm couples. Student's unpaired two-sided t test; ns: not significant. **B.** 5-AzaC significantly inhibits *S. mansoni* egg production. Thirty adult worm pairs were cultured either in the presence or absence of 491 µM 5-AzaC. Each culture condition was replicated (n = 5) and eggs were collected and counted after 48 hours. Mean ± SEM are shown. Student's unpaired two-sided t test was applied. *p-value = 0.02. **C.** Light microscopy of schistosome eggs laid by control worm pairs (control) or 5-AzaC treated worm pairs for 48 h (5-AzaC, 491µM). Scale bar: 100 µm.

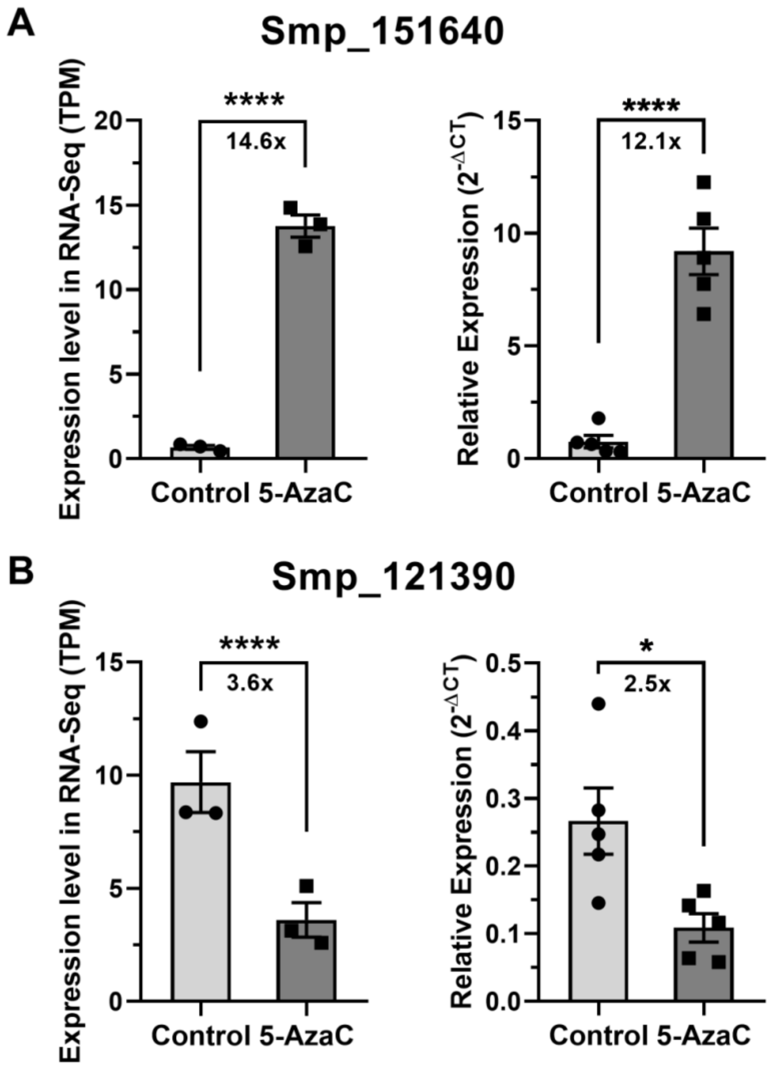

Supplementary Figure S5: Expression profiles in *S. mansoni* females of selected protein-coding genes differentially expressed after 5-AzaC treatment (491 µM). Two protein-coding genes were used as controls after re-analysis of RNA-Seq public datasets of 5-AzaC treated *S. mansoni* females from Geyer *et al*., 2018 [1] for validation by RT-qPCR in females. For each of the two protein-coding genes, their expression profiles in RNA-Seq are shown as TPM (transcripts per million) on the left, whereas the RT-qPCR results are shown on the right: **A**. Smp_151640 (*Insulin-like growth factor I*); **B**. Smp_121390 (*Genome polyprotein*). For the RNA-Seq data, three biological replicates were analyzed; the fold-changes and p-values represented by asterisks that are shown in the brackets were obtained using DESeq2. For the RT-qPCR data, mean ± SEM from five biological replicates are shown; *p<0.05, ****p<0.0001. Student's unpaired two-sided t test.

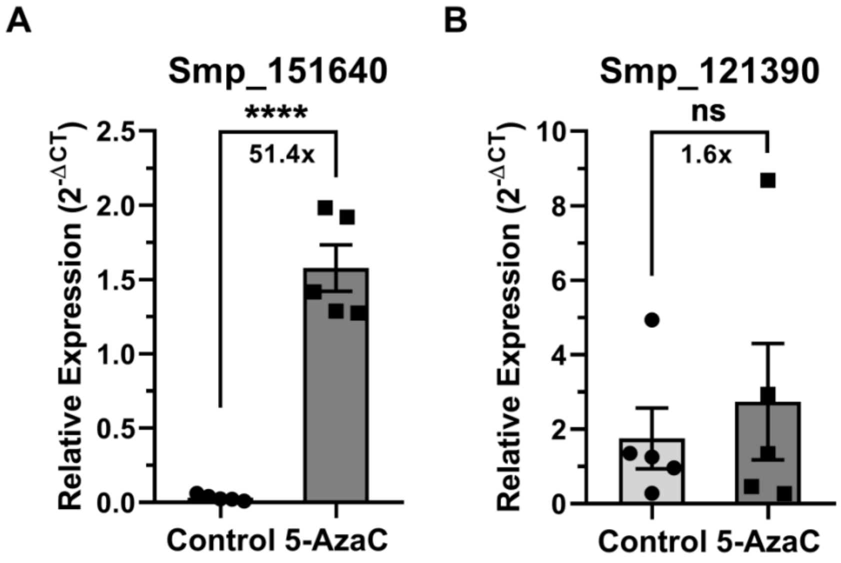

Supplementary Figure S6: Expression profiles in *S. mansoni* males of selected protein-coding genes differentially expressed after 5-AzaC treatment (491 µM). Two protein-coding genes were used as controls after re-analysis of RNA-Seq public datasets of 5-AzaC treated *S. mansoni* females from Geyer *et al*., 2018 [1] for evaluation of differential expression by RT-qPCR in males. For each of the two protein-coding genes, the expression profiles in controls and in 5-AzaC treated *S. mansoni* males by RT-qPCR are shown: **A**. Smp_151640 (*Insulin-like growth factor I*); **B**. Smp_121390 (*Genome polyprotein*). Mean ± SEM from five biological replicates are shown; Student's unpaired two-sided t test was applied. ****p<0.0001; ns: not significant.

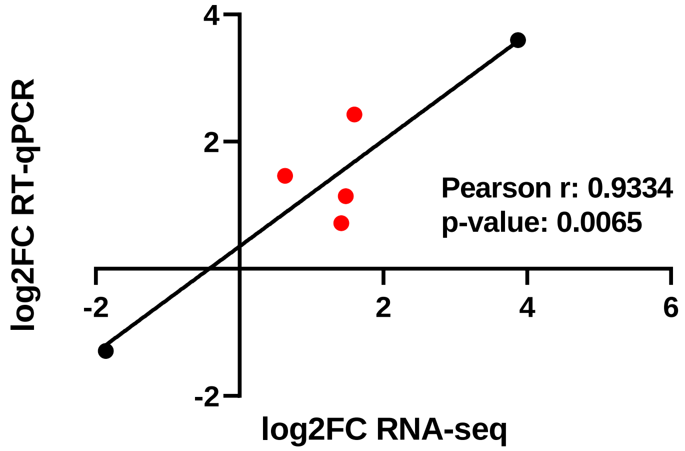

Supplementary Figure S7: Correlation between RNA-Seq and RT-qPCR analysis. Pearson correlation between the fold changes (FC) in expression measured by RNA-seq or RT-qPCR of six selected genes (four lincRNAs – red dots – and two protein-coding genes – black dots); fold changes were obtained by measuring the expression after treatment of females with 5-AzaC and comparing with expression in the control condition. Log2FC of the six genes obtained with the RNA-Seq assay is represented in the x-axis, and log2FC of the six genes obtained with RT-qPCR is represented in the y-axis.

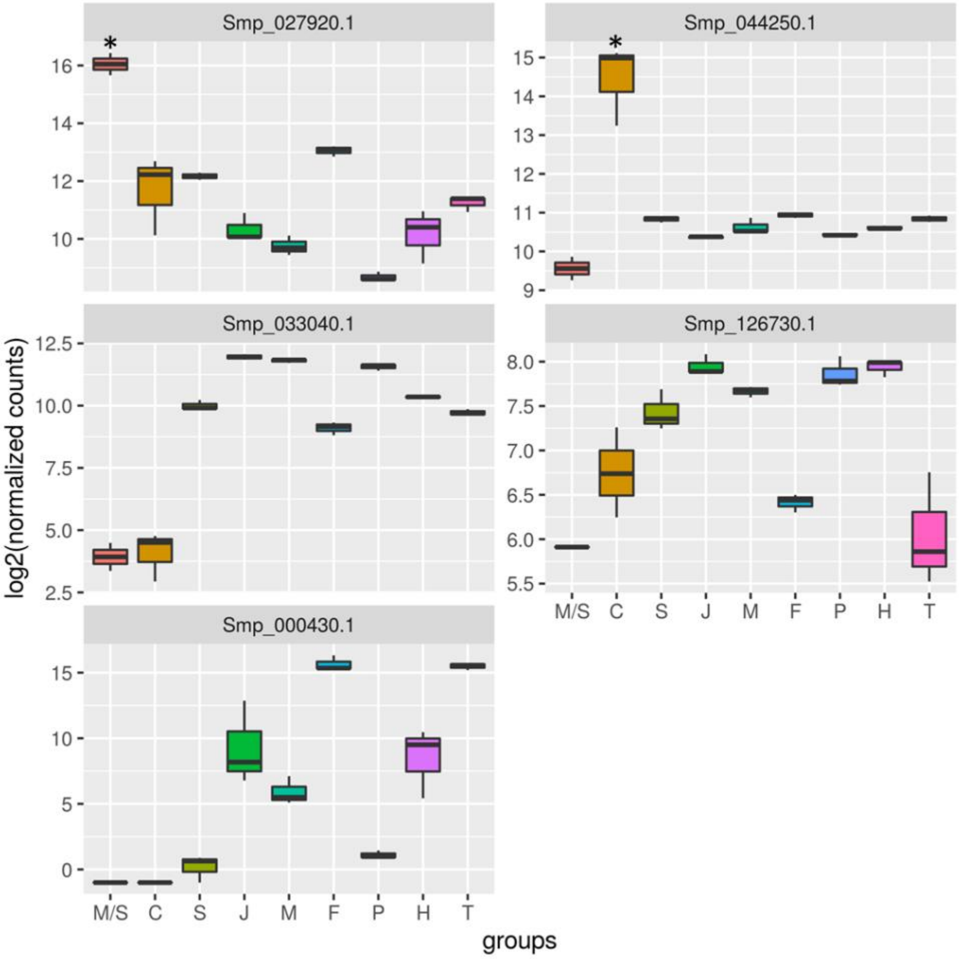

Supplementary Figure S8: RNA-seq expression levels in *different S. mansoni* stages of protein-coding genes used as sample markers. The expression levels (shown as log2 of normalized counts) of the five protein-coding genes whose gene IDs are indicated at the top of each panel are shown. The y-axis shows the expression level for each protein-coding gene in the RNA-seq assays (log2 of normalized counts) as determined at the stage indicated in the x-axis as follows: miracidia/sporocysts (M/S), cercariae (C), schistosomula (S), juveniles (J), adult males (M), adult females (F), posterior somatic tissues (P), heads (H) and tails (T). **A.** Smp_027920 (*Tubulin alpha-1 chain*, with high expression in eggs); **B**. Smp_044250 (*STAM-binding protein*, with high expression in cercariae); **C**. Smp_033040 (*L-lactate dehydrogenase A chain*, with high expression in schistosomula, juveniles and adult males); **D.** Smp_126730 (*5-hydroxytryptamine receptor 1A*, with high expression in juveniles and adult males); **E.** Smp_000430 (*Putative eggshell protein,* with high expression in adult females). Only transcripts that were upregulated in one stage/tissue when compared with all others were considered as significantly more expressed in that stage/tissue and are marked with an asterisk. *p-value < 0.05.

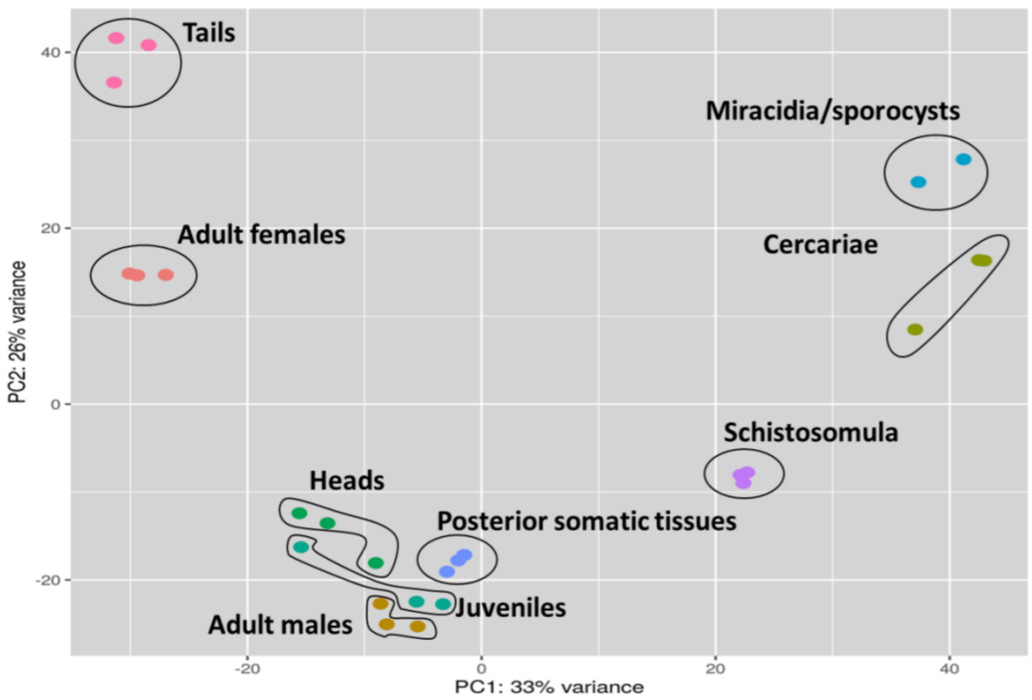

Supplementary Figure S9: Clustering of RNA-Seq biological replicates assessed by principal component analysis (PCA). RNA-Seq data from 26 public RNA-Seq libraries (listed in Supplementary Table S7) representing six life-cycle stages (miracidia/sporocysts, cercariae, schistosomula, juveniles, adult males, adult females) and adult worm heads, tails and posterior somatic tissues were re-analyzed using the *S. mansoni* genome PRJEA36577 (v.7) retrieved from WormBase and the recently published transcriptome that includes long non-coding RNAs [2] as reference. PCA plot was obtained after normalization using the vst function followed by the plotPCA function from DESeq2.

Supplementary References

1 Geyer, K. K. *et al.* The anti-fecundity effect of 5-azacytidine (5-AzaC) on Schistosoma mansoni is linked to dis-regulated transcription, translation and stem cell activities. *Int J Parasitol Drugs Drug Resist* **8**, 213-222, doi:10.1016/j.ijpddr.2018.03.006 (2018).

2 Maciel, L. F. *et al.* Weighted Gene Co-Expression Analyses Point to Long Non-Coding RNA Hub Genes at Different Schistosoma mansoni Life-Cycle Stages. *Front Genet* **10**, 823, doi:10.3389/fgene.2019.00823 (2019).

3 Lu, Z. *et al.* Schistosome sex matters: a deep view into gonad-specific and pairing-dependent transcriptomes reveals a complex gender interplay. *Sci Rep* **6**, 31150, doi:10.1038/srep31150 (2016).
